# Enduring relief or fleeting respite? Bitcoin as a hedge and safe haven for the US dollar

**DOI:** 10.1007/s10479-024-05884-y

**Published:** 2024-03-14

**Authors:** Thomas Conlon, Shaen Corbet, Richard McGee

**Affiliations:** 1 Smurfit Graduate School of Business, University College Dublin, Blackrock, Co. Dublin Ireland; 2 DCU Business School, Dublin City University, Dublin 9, Ireland; 3 School of Accounting, Finance and Economics, University of Waikato, Hamilton, New Zealand

**Keywords:** Bitcoin, Currency, Hedge, Safe haven, Wavelet, Quantile coherency, G10, G12

## Abstract

Can technology protect investors from extreme losses? This paper investigates the short- and long-run hedging and safe haven properties of Bitcoin for the US dollar over the period 2010–2023, incorporating the COVID-19-related market turmoil. Our findings reveal that (i) Bitcoin acts as a strong hedge for all US dollar currency pairs examined, (ii) Bitcoin functions as a weak safe haven for the US dollar at short investment horizons, as indicated by a limited relationship during acute negative price movements, (iii) Bitcoin, instead of acting as a safe haven may, instead, increase aggregate risk at long horizons during periods of extreme losses. The analysis, performed using a series of horizon-dependent econometric tests, provides evidence of some US dollar risk-reduction benefits from Bitcoin but limited potential for enduring relief from long-run extreme negative US dollar rate movements.

## Introduction

Traditional fiat currencies are subject to many risks, not least inflation, devaluation, and financial crises (Kaminsky, 2006; Borensztein & De Gregorio, 1999). Introduced as a technological alternative to fiat currencies, Bitcoin (BTC), the largest digital currency by market capitalisation, presents several characteristics that may help circumvent some of the weaknesses associated with fiat currencies. The supply of BTC is determined by an algorithm, limiting the opportunities for devaluation through inflation, and BTC is not linked to monetary policies (Yermack, 2015).^1^ As these characteristics are also common to gold, often held to protect wealth against unexpected market events, researchers have suggested that BTC may likewise act as a store of value during financial turmoil when investors seek refuge in so-called safe haven assets. In this paper, we assess whether BTC acts as a hedge or safe haven asset for US dollar (USD) exchange rates across a spectrum of different time horizons, inclusive of several crisis periods incorporating the European sovereign debt crisis, the COVID-19 pandemic, along with the subsequent period of elevated price inflation.

Evidence that gold has hedging and safe haven properties for the US dollar (USD) is extensive (Reboredo, 2013; Zagaglia & Marzo, 2013).^2^ While BTC might share some safe haven characteristics with gold, only a narrow set of literature has explored the possibility of BTC acting as a hedge or safe haven for currency. Urquhart and Zhang (2019) is the paper which is closest to ours, demonstrating that BTC has safe haven and hedging properties for currencies using hourly data from 2014 to 2017. Our paper differs in several important ways. First, we focus on the implications for the marginal investor at short- and long-horizons rather than examining intraday hedging and safe haven properties. This distinction is significant, as compelling evidence suggests that investors use one year as their evaluation horizon (Benartzi & Thaler, 1995). Second, we adopt several econometric methodologies appropriate to identify safe haven characteristics of BTC for currencies, especially at long horizons. These methodologies allow us to pinpoint the specific times, horizons, and market conditions under which any hedging and safe haven characteristics exist. Finally, our dataset runs to June 2023, incorporating the market turmoil associated with the COVID-19 pandemic, the first major global market crisis since the introduction of BTC, along with the subsequent period of elevated inflation.^3^ These phases provide a stringent test of the ability of BTC to act as a safe haven.

The dependence of financial characteristics on the interval, or horizon, at which they are estimated has been well documented. The estimation of systematic risk, index serial correlation, and higher-order moments such as skewness are well known to depend upon the measurement interval examined (Hawawini, 1980; Schwartz & Whitcomb, 1977; Levhari & Levy, 1977). More recent contributions have documented that asset pricing, mutual fund performance and benefits from international diversification are all conditional upon the time horizon assessed (Jin et al., 2023; Chaudhuri & Lo, 2019; Kamara et al., 2015; Rua & Nunes, 2009). To explain horizon-based financial characteristics, research has focused on differing clientele holding periods, trading frictions and asset serial covariance (Conlon et al., 2018a; Kamara et al., 2018; Cohen et al., 1983). Motivating our analysis, financial hedging properties, including hedge ratios and performance, have been shown to depend upon the horizon examined (Lien & Shrestha, 2007; In & Kim, 2006). Building on this literature, Bekiros et al. (2017) and Bredin et al. (2015) provide evidence consistent with different short- and long-run hedging and safe haven attributes for gold. In light of this previous evidence for contrasting features of financial time series at different horizons, any examination of BTC’s hedging and safe haven properties for currencies needs to account for distinctive horizon-based relationships.

To uncover the short- and long-run dependency structure between BTC and currencies, we utilize three different econometric approaches, each shedding light on a different aspect of the research question. First, using the regression approach proposed by Baur and McDermott (2010) and Baur and Lucey (2010) to test for hedging and safe haven characteristics, we assess the short- and medium-run interrelationships. Next, we employ the continuous wavelet transformation to simultaneously decompose the covariation between BTC and currencies over calendar time and frequency (or, interchangeably, time horizon). As safe haven properties are only found at points in calendar time associated with marked asset value losses, the use of wavelet-based tools provides an effective lens to identify the appropriateness of an asset as a hedge and safe haven across multiple horizons (Bredin et al., 2015). Finally, to establish the safe haven characteristics of BTC for extreme downside price movements in the USD currency rate, we use the quantile coherency approach proposed by Baruník and Kley (2019). This allows us to isolate the dependency between BTC and USD rates at short- and long-horizons for specific quantiles.

Our empirical findings support BTC’s capacity to act as a hedge against fluctuations in USD rates. A negative relationship between BTC and currencies, commensurate with strong hedging properties, is found on average. Wavelet coherency sheds further light on this relationship, highlighting that the hedging properties result from strong interdependence on irregular occasions alongside periods of limited interdependence. Moreover, during the COVID-19 market turmoil, the negative relationship is characterised by losses in BTC concurrent with gains for the USD, implying that BTC’s apparent strong hedging properties may result from positive currency returns aligned with negative BTC changes. Assessing the safe haven properties of BTC for USD exchange rates, we find limited evidence of dependency at short-run time horizons during times of turmoil. This indicates that BTC is a weak short-run safe haven for the USD. At longer horizons, however, we provide evidence that BTC occasionally experiences states of a significant positive relationship with changes in the USD exchange rates with EUR, CHF, and JPY, conditional upon the USD rate experiencing extreme losses. This indicates that an allocation to BTC would increase the magnitude of aggregate losses at exactly the juncture when long-run investors seek shelter from market turmoil.

Our paper makes various contributions to the literature. While our findings corroborate some previous results indicating short-run currency hedging and safe haven properties of BTC (Urquhart & Zhang, 2019), we contribute new empirical insights regarding the potential dangers of BTC for investors seeking long-run protection from extreme currency movements. As investors operate at heterogeneous horizons, the distinction between short- and long-run horizon properties of BTC in this paper allows us to investigate the extent to which hedging and safe haven properties are distributed across horizons. Previous research has also been constrained by the lack of a major worldwide market crisis since the introduction of BTC (Maghyereh & Abdoh, 2020; Urquhart & Zhang, 2019; Baumöhl, 2019). Our work builds on this by using an expansive data series that incorporates the market turmoil associated with the COVID-19 pandemic, providing the first crisis-period assessment of the short- and long-run currency hedging and safe haven properties of Bitcoin.^4^ Furthermore, the combination of methods allows for a clear identification of the hedging and safe haven properties associated with these horizons at particular points in calendar time and during significant market turbulence.

The relevancy of BTC as a hedge and safe haven for traditional assets has received some attention in recent years. Shahzad et al. (2019) indicate that BTC has been a weak safe haven for international equity indices since February 2018. Assessing the response of BTC returns to market shocks, Klein et al. (2018) demonstrate a lack of any associated safe haven or hedging capabilities for developed equity markets. Bouri et al. (2017), using a dynamic conditional correlation model, conclude that BTC has, at best, limited hedging and safe haven qualities across a range of asset classes. Cryptocurrency derivatives have also been analysed to investigate hedging capabilities (Akyildirim et al., 2021; Deng et al., 2021). Smales (2019) reflects upon attributes of BTC which impede its hedging and safe haven capacity, including market volatility, liquidity, and transaction costs. Focusing on the equity bear market associated with the COVID-19 pandemic, Conlon and McGee (2020) demonstrate that allocations to BTC increase portfolio downside risk. Conlon et al. (2020) assess a range of cryptocurrencies across international markets, finding only limited evidence of any safe haven potential during the COVID-19 downturn. Maghyereh and Abdoh (2020) focus on the tail dependency between BTC and various asset classes, providing evidence of a negative tail link between BTC and the USD-EUR rate at a monthly horizon but no links at longer horizons. The distinction with our findings results from an extended data set incorporating the COVID-19 crisis, alongside a focus on different hedging horizons. Baumöhl (2019), examining a dataset concluding in December 2017, also suggests a contrasting negative long-run tail relationship between cryptocurrencies and currencies, which is found to have reversed once the turmoil associated with the COVID-19 crisis is incorporated. A similar focus on the pandemic and cryptocurrency interaction was presented by Ftiti et al. (2021), while other episodes of price explosivity and market interactions have also been considered (Koutmos, 2020; Cai et al., 2021; Ben Omrane et al., 2021; Huynh et al., 2021; Cretarola & Figà-Talamanca, 2021).

One explanation for BTC not comprehensively fulfilling its expected role as a hedge or safe haven might be the well-documented market inefficiency issues surrounding the asset (Urquhart, 2017; Bariviera, 2017; Urquhart, 2016). Further issues with regards to illicit behaviour, Cybercriminality, gambling and irregular exchange dynamics also provide explanatory value with regards to not just the differential behaviour of cryptocurrency markets in comparison to traditional asset markets but the rapidly changing dynamics and volatility inherent therein (Cioroianu et al., 2020; Corbet et al., 2020a; Conlon & McGee, 2019). While cryptocurrency markets present evidence of disconnect and, therefore, diversification potential, market participation by speculative investors may result in adverse price movements and liquidity risks during downturns (Griffin & Shams, 2020; Fry, 2018). The hedging benefits found on average are not assured during common severe price movements across markets, restricting BTC’s role as a safe haven. The inflation hedging properties have also been examined empirically, with divergent inference dependent on the methodology invoked (Blau et al., 2021; Conlon et al., 2021). While we find limited evidence of safe haven properties in recent times, most notably during the recent period of elevated inflation, this may evolve alongside developing market maturity and efficiency improvement, particularly through the addition of derivatives markets (Nan & Kaizoji, 2019; Baur & Dimpfl, 2019; Kapar & Olmo, 2019; Hu et al., 2020).

The paper is laid out as follows. Section 2 describes the methodologies employed in the paper, while the relevant data is outlined in Sect. 3. The empirical findings are described and discussed in Sects. 4, and 5 concludes.

## Methodology

To identify BTC’s hedging and safe haven characteristics for currencies, we employ a series of sophisticated econometric tests. Section 2.1 describes a baseline non-linear regression model to identify hedging and safe haven properties for BTC. In Sect. 2.2, we briefly describe the continuous wavelet transformation and define the wavelet squared coherency, a measure allowing for a localized assessment of the horizon-dependent interrelationships between two time series. The quantile cross-spectral coherency is outlined in Sect. 2.3, providing a framework to examine interrelationships between BTC and currencies at different horizons during periods of turmoil.

The formal definitions of a hedge and safe haven in this paper follow those proposed by Baur and McDermott (2010) and Baur and Lucey (2010) and are given as:

- *A strong (weak) hedge is an asset that is negatively correlated (uncorrelated) with another asset on average.*
- *A strong (weak) safe haven is an asset that is negatively correlated (uncorrelated) with another asset in times of market stress or turmoil.*

### Regression analysis

We first test the hedging and safe haven properties of BTC using the econometric approach proposed by Baur and McDermott (2010) and Baur and Lucey (2010) for the case of gold. The model is given by

1a $$\begin{aligned} r_{btc,t}= & {} \alpha + \beta _1 r_{c,t} + \beta _2^{5\%} r_{c,t} Q_5 + \beta _2^{2.5\%} r_{c,t} Q_{2.5} + \beta _2^{1\%} r_{c,t} Q_1 + \gamma ^{5\%} Q_5 \nonumber \\{} & {} \quad + \gamma ^{2.5\%} Q_{2.5} + \gamma ^{1\%} Q_1 + e_t \end{aligned}$$

1b $$\begin{aligned} h_t= & {} \omega + a e^2_{t-1} + b h_{t-1}, \end{aligned}$$

where $r_{btc,t}$ and $r_{c,t}$ are the returns of BTC and the currency pair to be examined. The coefficient on $r_{c,t}$ captures the hedging capacity of BTC, where a coefficient insignificantly different from zero represents hedging possibilities, and a negative and significant coefficient indicates strong hedging potential. $Q_1$, $Q_{2.5}$ and $Q_5$ are dummy variables taking value one if $r_{btc,t}$ is below the $1\%$, $2.5\%$ and $5\%$ quantile and zero otherwise. The parameters $\beta _2^{1\%}$, $\beta _2^{2.5\%}$ and $\beta _2^{5\%}$ capture any relationship between BTC and currencies at the lower tail. The total effect is a sum of the relevant coefficients as, if the currency return exceeds a certain threshold, it also exceeds all larger thresholds. For example, if returns exceed the $2.5\%$ quantile, they also exceed the $5\%$ quantile. If the coefficients are negative and significantly different than zero, this indicates that BTC is a strong safe haven for currency. If coefficients are insignificantly different from zero, BTC is a weak safe haven for currency.

Equation 1b is a GARCH(1,1) model used to account for the presence of heteroscedasticity in the data. The models are jointly estimated using Maximum Likelihood. In the analysis below, the models are estimated on data measured at various frequencies through sub-sampling of daily data at longer horizons. The analysis is limited, however, by a reduction in the quantities of available data at longer horizons. To facilitate analyses at long horizons of interest to investors, we build on this preliminary analysis using methods that can estimate long-horizon relationships without sub-sampling data.

### Wavelet squared coherency

In this paper, we seek to identify the points in calendar time at which two time series are interrelated to determine whether any relationships uncovered are horizon-dependent and to detect their direction. This allows us to quantify the strength of the hedging potential of BTC at all points in calendar time and the safe haven capacity during times of turmoil in currency markets.

Wavelet analysis allows for a novel, time-dependent perspective on the hedging properties of BTC. As highlighted by Bredin et al. (2015) in the case of gold, wavelet analysis has the unique capacity to trace hedging properties across three dimensions simultaneously. First, wavelet coherency is locally estimated at each point, allowing us to assess the dynamic interactions between BTC and currencies. Second, wavelet analysis allows us to understand the horizon or frequency dependence of the hedging properties. This is critical, as many financial characteristics change markedly at different horizons, and a long-run investor may encounter different risks to their short-run counterparts. Finally, the coherency plots that we provide show the direction of the relationship and whether there are any lead-lag effects between currencies and BTC. Combined with the previous properties, this allows us to simultaneously identify the extent and direction of any hedging properties at specific points in time for a range of horizons.

The wavelet transformation provides a variance decomposition of a time series over both time and scale. While this resembles the Fourier transformation, wavelets present better localization properties and can accommodate non-stationary behaviour. The wavelet transformation provides particular benefits for financial time series, especially during extreme volatility and localised discontinuities. The use of wavelets in a financial context is originally due to Ramsey et al. (1995) but has been applied to uncover time-scale properties in various contexts,^5^

In this paper, the continuous wavelet transformation is used to decompose time series variation across time and horizon and to determine the level and direction of coherency between pairs of time series. This framework is based on Torrence and Compo (1998) and Grinsted et al. (2004). The continuous wavelet transformation has been applied to understand the co-dependence structure between financial and economic time series on numerous occasions.^6^

The wavelet transformation utilizes a “small wave” or wavelet, $\psi (t)$, which is a function of a time parameter *t*. For time series *x*(*t*), expressed over the interval $[-\alpha<t<\alpha ]$, the associated wavelet coefficients $W\left( \tau , \epsilon \right)$ are calculated using:

2 $$\begin{aligned} W\left( \tau , \epsilon \right) = \sum _{t=1}^{N} x\left( t\right) \psi ^{*}\left[ \frac{t-\tau }{\epsilon }\right] , \end{aligned}$$

where $[\epsilon >o;-\alpha<\tau <\alpha ]$ and $\psi ^{*}$ is the complex conjugate of the wavelet. $\epsilon$ and $\tau$ define the scale associated with the transformation and location of the window, respectively, while $\frac{1}{\epsilon }$ is a normalization factor.

The Morlet wavelet basis is selected for this study due to its strong localization properties.^7^ The calculation of the Morlet wavelet is based upon the product of a sine curve with a Gaussian and written as:

3 $$\begin{aligned} \psi (t) = \pi ^{\frac{1}{4}}\left( e^{i \omega _0 t} - e^{-\frac{\omega _0^2}{2}}\right) e^{\frac{-t^2}{2}}. \end{aligned}$$

The wavenumber $\omega _0$ controls the number of oscillations within the Gaussian envelope. Following Grinsted et al. (2004), we set $\omega _0 = 6$, as this provides a wavelet scale which is almost equal to the Fourier period, aiding interpretation. Setting $\omega _0 = 6$, the Morlet wavelet can be written as:

4 $$\begin{aligned} \psi (t) = \pi ^{\frac{1}{4}} e^{i \omega _0 t} e^{\frac{-t^2}{2}}. \end{aligned}$$

Using the coefficients, $W\left( \tau ,\epsilon \right)$, emerging from the wavelet transformation, we can form a number of metrics which allow for a time-scale understanding of financial time series. The spectral energy or variance of a time series can be characterised over both time and scale (frequency) using the wavelet power spectrum, providing information relating to the variance of a single time series for a particular scale at a given point in time.^8^ The power spectrum for a time series *x*(*t*) is given by $|W_{\epsilon ,\tau }^2(x)|$, the square of the wavelet coefficient at scale $\epsilon$ and location $\tau$.

For two time series, the cross-wavelet power spectrum provides insights relating to the common variation over time and scale. This is calculated using the product of the wavelet coefficients $W_{\epsilon ,\tau } (r,s) = W_{\epsilon ,\tau } (r) * W_{\epsilon ,\tau } (s)$, where $*$ is defined as a complex conjugate. As the cross-power spectrum may be affected by differences in variance from the two time series, the wavelet squared coherency is often examined in practice. This is given by normalizing the smoothed cross-wavelet spectrum by the associated smoothed wavelet power spectrum:

5 $$\begin{aligned} \rho ^2_{\epsilon ,\tau } = \frac{|Q\left( \epsilon ^{-1} W_{\epsilon ,\tau }(r,s)\right) |^2}{Q\left( |\epsilon ^{-1}W_{\epsilon ,\tau }(r)\right) |^2Q\left( |\epsilon ^{-1}W_{\epsilon ,\tau }(s)\right) |^2}. \end{aligned}$$

where *Q* is the smoothing operator in both time and scale (Torrence & Compo, 1998). Wavelet coherency can be considered a squared correlation, providing a measure of co-variation between two series divided by their variation at different scales and points in time. Squared coherency, $\rho ^2_{\epsilon ,\tau }$, is bounded between zero (no comovement) and one (perfect comovement). Monte-Carlo methods are applied to determine the region of statistical significance for the wavelet coherency (Aguiar-Conraria & Soares, 2014; Torrence & Compo, 1998). To determine the direction of the relationship between two time series and any evidence of lead-lag effects, we use the wavelet multi-scale phase (Aguiar-Conraria & Soares, 2014). For two time series *x*(*t*) and *y*(*t*) this is given by:

6 $$\begin{aligned} \theta _{\epsilon ,\tau } \left( x,y\right) = tan^{-1} \left( \frac{\Im \lbrace Q\left( \epsilon ^{-1} W_{\epsilon ,\tau }(x,y)\right) \rbrace }{\Re \lbrace Q\left( \epsilon ^{-1} W_{\epsilon ,\tau }(x,y)\right) \rbrace } \right) . \end{aligned}$$

$\Re$ and $\Im$ correspond to the real and imaginary components of the wavelet coefficients, respectively, and *Q* is the smoothing parameter. In a plot of wavelet coherency, phase arrows are used to indicate the direction of co-movement and any lead-lag effects between the two time series under examination. East (west) facing arrows represent the in- (out-of-) phase, while north (south) facing arrows indicate that time series two leads (lags) time series one. A north-east (south-east) facing arrow symbolizes that the series is in phase but that time series two (time series one) leads to time series one (time series two). A north-west (south-west) facing arrow signifies that the series are out-of-phase but that time series one (time series two) leads time series two (time series one). Further details regarding the interpretation of phase arrows can be found in Funashima (2017).

### Quantile cross-spectral coherency

While wavelet coherency allows us to decipher the points in calendar time at which BTC acts as a hedge or safe haven for currencies, this analysis does not provide a direct estimate of the strength of any relationships during extreme market disturbances. For this reason, we also employ the quantile cross-spectral coherency proposed by Baruník and Kley (2019), which allows us to quantify the safe haven properties of BTC during periods of acute losses in currency markets.^9^ Given a strictly stationary time series, *x*(*t*), we denote the marginal distribution function by $F_x$. The corresponding quantile function is then given by $q_x\left( \tau \right) = F^{-1}_x \left( \tau \right) = inf\{q \in {\mathbb {R}}: \tau \le F_x(q)\}$, where the quantile $\tau \in [0,1]$. The quantile coherency between strictly stationary time series, *x*(*t*) and *y*(*t*), can be written as:

7 $$\begin{aligned} \mathfrak {R}^{x,y}\left( \omega ; \tau _1, \tau _2\right) = \frac{\mathfrak {f}^{x,y}\left( \omega ; \tau _1, \tau _2\right) }{\left( \mathfrak {f}^{x,x}\left( \omega ; \tau _1, \tau _1\right) \mathfrak {f}^{y,y}\left( \omega ; \tau _2, \tau _2\right) \right) ^{\frac{1}{2}}}, \end{aligned}$$

$\omega \in \mathbb {R}$ and $\tau _{1,2} \in \left[ 0,1\right]$. $\mathfrak {f}^{x,y}\left( \omega ; \tau _1, \tau _2\right)$ is the quantile cross-spectral density kernel, estimated using the Fourier transformation,

8 $$\begin{aligned} \mathfrak {f}^{x,y}\left( \omega ; \tau _1, \tau _2\right) = \frac{1}{2\pi }\sum _{k = -\infty }^{\infty } \gamma _k^{x,y}\left( \tau _1, \tau _2\right) e^{-ik\omega }. \end{aligned}$$

$\gamma _k^{x,y}\left( \tau _1, \tau _2\right)$ is, itself, a quantile cross-covariance kernel, given by:

9 $$\begin{aligned} \gamma _k^{x,y}\left( \tau _1, \tau _2\right) = cov\Big (I \{ X_{t+k} \le q_{x} \left( \tau _1\right) \}, I\{Y_{t} \le q_{y} \left( \tau _2\right) \} \Big ), \end{aligned}$$

where $I\{D\}$ is an indicator function for an event *D*, $k\in \mathbb {Z}$ and $\tau _{1,2} \in \left[ 0,1\right]$. Varying *k* allows us to determine the serial dependence between time series *x*(*t*) and *y*(*t*).

The estimator for the quantile cross-spectral density is called the copula cross-periodogram (CCR). To ensure the consistency of the CCR periodograms, the quantile cross-spectral density is smoothed across frequencies. A detailed treatment of properties of the quantile coherency may be found in Baruník and Kley (2019).

## Data

End-of-day currency rates data are obtained from Refinitiv Eikon. As BTC prices are quoted in US dollar terms, we examine links between spot currency rates relative to the US dollar as a baseline for BTC’s hedging and safe haven properties for currencies. Specifically, we consider cross-rates between the US dollar (USD) and Euro (EUR), Pounds Sterling (GBP), Swiss Franc (CHF), Canadian dollar (CAD), Japanese Yen (JPY) and Australian dollar (AUD). These currencies are selected as they account for eight of the nine largest trading volumes by value.^10^ BTC data are obtained from Coinmetrics, using the CM reference rates formed using a methodology which adheres to the International Organisation of Securities Commissions (IOSCO) framework of principles for financial market benchmarks. All data are daily, and logarithmic returns are examined over a period stretching from July 19th 2010 to June 30th 2023, a total of 3379 daily returns. To allay potential problems in respect of limited trading of Bitcoin in the period after its release, we also examine a period from January 1st 2016 to June 30th 2023, a total of 1,956 daily returns. Finally, as indicated earlier, Bitcoin has been proposed as a hedge against inflation. To test whether this impacts our findings, we examine the period after the initial COVID-19 shock, when inflation surged to levels not seen for decades. Specifically, we examine the period July 1st 2020 to June 30th 2023, a total of 783 daily returns.

Summary statistics for the time series over each period examined are presented in Table 1. The mean and standard deviation for BTC is vastly greater than that observed for any of the currencies across all periods, raising some initial questions about its ability to act as a hedge. Over the full period, the USD-CHF exchange rate has the lowest skewness and highest kurtosis of the currencies considered. This is partially attributable to its minimum daily return of $-17.1\%$ (15th January 2015). CHF is also the only currency in which the USD depreciates respectively over the period examined, showing cumulative returns of $-16.2\%$.

**Table 1:** Summary statistics

	BTC	EUR	GBP	CHF	CAD	JPY	AUD
*2010–2023*							
Mean	0.950	0.013	0.013	$-$ 0.012	0.017	0.038	0.020
Standard Deviation	0.906	0.083	0.092	0.095	0.076	0.090	0.104
Skewness	$-$ 0.522	$-$ 0.011	0.794	$-$ 1.527	0.058	$-$ 0.202	0.225
Kurtosis	17.558	4.778	17.574	57.130	4.233	8.587	5.215
Minimum	$-$ 0.665	$-$ 0.026	$-$ 0.032	$-$ 0.114	$-$ 0.020	$-$ 0.039	$-$ 0.033
Cumulative Returns	12.841	0.173	0.179	$-$ 0.162	0.227	0.509	0.264
Var 99%	0.170	0.014	0.015	0.015	0.012	0.015	0.016
Var 99% January -June 2020	0.125	0.016	0.019	0.016	0.034	0.023	0.029
*2016–2023*							
Mean	0.545	$-$ 0.001	0.019	$-$ 0.014	$-$ 0.006	0.023	0.011
Standard Deviation	0.713	0.073	0.102	0.073	0.074	0.089	0.100
Skewness	$-$ 0.684	$-$ 0.020	1.009	$-$ 0.343	$-$ 0.063	$-$ 0.507	0.245
Kurtosis	12.472	4.815	19.292	5.904	4.432	9.365	5.065
Minimum	$-$ 0.471	$-$ 0.022	$-$ 0.032	$-$ 0.032	$-$ 0.020	$-$ 0.039	$-$ 0.029
Cumulative Returns	4.262	$-$ 0.004	0.148	$-$ 0.112	$-$ 0.045	0.182	0.089
Var 99%	0.132	0.013	0.015	0.013	0.012	0.016	0.016
*2020–2023*							
Mean	0.384	0.009	$-$ 0.009	$-$ 0.018	$-$ 0.008	0.093	0.011
Standard Deviation	0.655	0.076	0.096	0.079	0.072	0.089	0.110
Skewness	$-$ 0.381	0.021	$-$ 0.001	$-$ 0.476	$-$ 0.091	$-$ 0.860	0.234
Kurtosis	7.309	4.281	5.754	6.303	3.611	9.822	3.705
Minimum	$-$ 0.264	$-$ 0.018	$-$ 0.030	$-$ 0.032	$-$ 0.020	$-$ 0.039	$-$ 0.024
Cumulative Returns	1.204	0.029	$-$ 0.029	$-$ 0.057	$-$ 0.025	0.291	0.034
Var 99%	0.117	0.013	0.016	0.014	0.011	0.016	0.016

Summary statistics are detailed for bitcoin and each of the currency return time series considered over three periods, 2010–2023, 2016–2023 and 2020–2023

The currency series considered are the US dollar (USD) exchange rates with the Euro (EUR), Pounds Sterling (GBP), Swiss Franc (CHF), Canadian dollar (CAD), Japanese Yen (JPY) and Australian dollar (AUD). Mean and standard deviation are given in annualized terms. VaR 99% and Var 99% January–June 2020, correspond to the historical simulation value at risk at a 99% confidence interval over the entire period and between January and June 2020, respectively

Table 1 also details daily downside risk statistics for each asset, estimated using non-parametric historical simulation value at risk (VaR) at a 99% confidence interval. BTC has a substantially greater level of downside risk than any of the currency pairs. AUD has the greatest estimated VaR from 2010–2023 among the currencies analysed. We observe an increase in VaR during the COVID-19 market turmoil for all currencies, suggesting that investors might look for hedging and safe haven instruments during this period.

Similar findings are evident over the other sub-periods examined. Bitcoin has the highest mean return and standard deviation and the lowest daily returns. The USD depreciated relative to the EUR, CHF and CAD over the period 2016–2023, while it is found to have depreciated relative to the GBP, CHF and CAD over the 2020–2023 interval. Throughout both periods, Bitcoin retains a VaR that is almost a magnitude larger than that of other currencies.

## Empirical findings

We examine the hedging and safe haven properties of BTC for currencies from a variety of different perspectives. First, in Sect. 4.1, we examine the ability of BTC to act as a hedge and safe haven for currencies using a traditional non-linear regression framework. Next, this analysis is expanded to place a stronger emphasis on the localized hedging and safe haven properties in time and scale, Sect. 4.2. Finally, Sect. 4.3 assesses the safe haven properties at extreme distributional quantiles for a series of different time horizons.

### Regression analysis

The ability of BTC to act as a hedge and safe haven for currencies is first examined using a regression analysis following the approach of Baur and Lucey (2010) and Baur and McDermott (2010). Results, detailed in Table 2, are presented over the three periods indicated earlier: 2010–2023, 2016–2023 and 2020–2023. To interpret the safe haven possibilities indicated by the regression, we need to consider the combination of coefficients. For example, the feasibility of BTC acting as a safe haven when the currency return is beyond the 2.5% threshold is given by summing the coefficients of $\beta _1$, $\beta _2^{5\%}$ and $\beta _2^{2.5\%}$.

**Table 2:** Regression testing the role of Bitcoin as a hedge and a safe haven for currencies

	2010–2023			2016–2023			2020–2023		
	Coeff	Std Er	t-Stat	Coeff	Std Er	t-Stat	Coeff	Std Er	t-Stat
Euro									
$\beta _1$	$-$ 0.47***	0.14	$-$ 3.33	$-$ 0.54**	0.23	$-$ 2.39	$-$ 1.57***	0.31	$-$ 5.14
$\beta _1 \, + {\, \beta _2^{5\%}}$	$-$ 0.49	9.51	$-$ 0.05	$-$ 16.80***	9.06	$-$ 1.85	15.36	30.90	0.50
${\beta _1 \, +} {\, \beta _2^{5\%}}$ ${+ \, \beta _2^{2.5\%}}$	2.74	5.60	0.49	4.49	6.87	0.65	16.23	14.80	1.10
${\beta _1 \, +} {\, \beta _2^{5\%}}$ ${+ \, \beta _2^{2.5\%}}$ ${+ \, \beta _2^{1\%}}$	$-$ 1.41	1.24	$-$ 1.13	1.51	10.34	0.15	18.76	18.80	1.00
Pounds sterling									
$\beta _1$	$-$ 0.77***	0.13	$-$ 5.83	$-$ 0.68***	0.15	$-$ 4.53	$-$ 1.49***	0.21	$-$ 7.24
$\beta _1 \, + {\, \beta _2^{5\%}}$	6.30	8.91	0.71	6.74	13.40	0.50	6.75	20.33	0.33
${\beta _1 \, +} {\, \beta _2^{5\%}}$ ${+ \, \beta _2^{2.5\%}}$	4.46	7.52	0.59	$-$ 0.53	6.91	$-$ 0.08	$-$ 7.26	7.71	$-$ 0.94
${\beta _1 \, +} {\, \beta _2^{5\%}}$ ${+ \, \beta _2^{2.5\%}}$ ${+ \, \beta _2^{1\%}}$	$-$ 0.06	1.37	$-$ 0.04	0.43	1.74	0.25	0.56	4.08	0.14
Swiss Franc									
$\beta _1$	$-$ 0.48***	0.13	$-$ 3.58	$-$ 0.53**	0.22	$-$ 2.41	$-$ 0.97***	0.29	$-$ 3.35
$\beta _1 \, + {\, \beta _2^{5\%}}$	0.08	4.05	0.02	4.61	23.18	0.20	$-$ 5.46	15.98	$-$ 0.34
${\beta _1 \, +} {\, \beta _2^{5\%}}$ ${+ \, \beta _2^{2.5\%}}$	$-$ 3.28	4.21	$-$ 0.78	12.60	23.29	0.54	26.06	19.88	1.31
${\beta _1 \, +} {\, \beta _2^{5\%}}$ ${+ \, \beta _2^{2.5\%}}$ ${+ \, \beta _2^{1\%}}$	1.22	1.21	1.01	11.95	22.58	0.53	11.36	14.08	0.81
Canadian dollar									
$\beta _1$	$-$ 1.00***	0.14	$-$ 7.12	$-$ 1.15***	0.19	$-$ 6.08	$-$ 3.10***	0.28	$-$ 11.14
$\beta _1 \, + {\, \beta _2^{5\%}}$	1.82	8.85	0.21	3.45	8.24	0.42	27.48	41.70	0.66
${\beta _1 \, +} {\, \beta _2^{5\%}}$ ${+ \, \beta _2^{2.5\%}}$	$-$ 4.13	13.14	$-$ 0.31	$-$ 9.62	19.39	$-$ 0.50	$-$ 35.88***	21.28	$-$ 1.69
${\beta _1 \, +} {\, \beta _2^{5\%}}$ ${+ \, \beta _2^{2.5\%}}$ ${+ \, \beta _2^{1\%}}$	3.81	2.81	1.36	0.46	4.66	0.10	$-$ 3.29	15.14	$-$ 0.22
Japanese Yen									
$\beta _1$	$-$ 0.09	0.14	$-$ 0.61	$-$ 0.20	0.20	$-$ 1.02	$-$ 0.35	0.29	$-$ 1.20
$\beta _1 \, + {\, \beta _2^{5\%}}$	$-$ 2.69	5.93	$-$ 0.45	$-$ 10.80	7.90	$-$ 1.37	6.46	11.78	0.55
${\beta _1 \, +} {\, \beta _2^{5\%}}$ ${+ \, \beta _2^{2.5\%}}$	2.13	5.45	0.39	9.41***	5.65	1.67	14.83***	8.57	1.73
${\beta _1 \, +} {\, \beta _2^{5\%}}$ ${+ \, \beta _2^{2.5\%}}$ ${+ \, \beta _2^{1\%}}$	$-$ 1.95***	0.91	$-$ 2.13	$-$ 2.80***	0.93	$-$ 3.03	$-$ 2.39	2.22	$-$ 1.07
Australian dollar									
$\beta _1$	$-$ 0.68***	0.11	$-$ 5.92	$-$ 0.77***	0.15	$-$ 5.22	$-$ 1.35***	0.18	$-$ 7.52
$\beta _1 \, + {\, \beta _2^{5\%}}$	$-$ 5.99	5.44	$-$ 1.1	4.08	7.85	0.52	1.03	26.20	0.04
${\beta _1 \, +} {\, \beta _2^{5\%}}$ ${+ \, \beta _2^{2.5\%}}$	$-$ 5.28	7.37	$-$ 0.72	0.37	11.11	0.03	$-$ 11.68	22.80	$-$ 0.51
${\beta _1 \, +} {\, \beta _2^{5\%}}$ ${+ \, \beta _2^{2.5\%}}$ ${+ \, \beta _2^{1\%}}$	$-$ 2.44	2.32	$-$ 1.05	$-$ 1.33	2.85	$-$ 0.47	$-$ 9.49	16.98	$-$ 0.56

This table reports coefficients associated with a regression model with GARCH error terms to assess the hedge and safe haven properties of Bitcoin

The estimated model is given by: $r_{btc,t} = \alpha + \beta _1 r_{c,t} + \beta _2^{5\%} r_{c,t} Q_5 + \beta _2^{2.5\%} r_{c,t} Q_{2.5} + \beta _2^{1\%} r_{c,t} Q_1 + \gamma ^{5\%} Q_5 + \gamma ^{2.5\%} Q_{2.5} + \gamma ^{1\%} Q_1 + e_t$ where $h_t = \omega + a e^2_{t-1} + b h_{t-1}$

The model is estimated for daily data for the USD exchange rate to currencies including the Euro (EUR), Pound Sterling (GBP), Swiss Franc (CHF), Canadian Dollar (CAD), Japanese Yen (JPY) and Australian Dollar (AUD)

Three periods are examined, July 2010–June 2023, January 2016–June 2023 and July 2020–June 2023

***, ** and * indicate statistical significance at the 1%, 5% and 10% levels respectively

Focusing first on the hedging properties of BTC for currencies, we examine the coefficient associated with $\beta _1$. For all periods, we find a negative and significant coefficient associated with $\beta _1$ for all currencies except JPY, indicating that BTC is a strong short-run hedge for all currencies. For JPY, the coefficient is not found to be significantly different from zero, indicating that BTC acts as a weak short-run hedge. The size of the coefficient varies from $-0.47$ (EUR) to $-1.00$ (CAD) over the entire period, indicating that, for a 1% move in the currencies, bitcoin is expected to change by between $-\,0.47\%$ and $-1\%$. The magnitude of the coefficient is found to be larger over the 2020 to 2023 period, with statistically significant coefficients ranging from $-0.97$ (CHF) to $-3.10$ (CAD). During this period of significant inflationary pressures, BTC acted as a strong hedge against adverse moves in the US Dollar.

Examining the coefficients associated with $\beta _2$, we provide evidence as to the ability of BTC to act as a safe haven when currency returns are below the $1\%$, $2.5\%$ and $5\%$ thresholds. In most cases, we find no support for a significant relationship between BTC and currencies, implying that BTC is a weak safe haven. Over the full period, the only evidence of BTC having strong, safe haven properties is for the USD-JPY rate at a $1\%$ threshold. The evidence persists over the 2016–2023 period but is not present during 2020–2023. Over the former period, Bitcoin is a strong safe haven for EUR, but only at a $5\%$ threshold. Over the most recent period, Bitcoin is found to be a strong safe haven for CAD but only at the $2.5\%$ level and not at a $5\%$ or $1\%$ level, highlighting a level of inconclusiveness in the safe haven implications. During the COVID period, policymakers adopted monetary and fiscal policies to respond to the rapid economic downturn. While some commonality existed in these policies, such as unconventional monetary policies, there were differences in the extent of the reaction (Benmelech & Tzur-Ilan, 2020). This, in turn, resulted in differences in the currency responses to the downturn, perhaps helping to explain the diversity in safe haven properties.

For the JPY, we find some evidence of a positive relationship with BTC when the latter experiences large losses. At the $5\%$ threshold, we find a positive coefficient during the 2016–2023 and 2020–2023 periods. However, this coefficient does not carry over to higher or lower thresholds, indicating that these findings may be a consequence of a small number of particular observations.

These baseline findings indicate strong currency hedging properties from BTC, acting as a strong hedge for most currencies assessed. We find no conclusive evidence that BTC acts as a strong safe haven for currency losses. In contrast to Urquhart and Zhang (2019), where BTC was found to act as a diversifier at an hourly horizon, our findings indicate that BTC provides weak safe haven properties during times of market stress.

#### Regression analysis with alternative horizons

Currency traders and hedgers may be interested in alleviating risk at longer horizons. To assess the hedging and safe haven potential of BTC for the USD at longer horizons, we carry out a further regression analysis. Table 3 documents the hedging and safe haven properties using one- and two-week returns data, employing the regression model defined in Eq. 1.

**Table 3:** Regression testing the role of Bitcoin as a hedge and a safe haven for currencies

	Weekly			Two week		
	Coeff	Std Er	t-Stat	Coeff	Std Er	t-Stat
Euro						
$\beta _1$	$-$ 1.18***	0.42	$-$ 2.83	$-$ 0.79	0.59	$-$ 1.34
$\beta _1 \, + {\, \beta _2^{5\%}}$	$-$ 10.78	83.55	$-$ 0.13	36.74	272.41	0.13
${\beta _1 \, +} {\, \beta _2^{5\%}}$ ${+ \, \beta _2^{2.5\%}}$	33.78	67.90	0.50	$-$ 11.90	77.70	$-$ 0.15
${\beta _1 \, +} {\, \beta _2^{5\%}}$ ${+ \, \beta _2^{2.5\%}}$ ${+ \, \beta _2^{1\%}}$	23.41	55.84	0.42	0.07	54.98	0.00
Pounds sterling						
$\beta _1$	$-$ 1.45***	0.43	$-$ 3.36	$-$ 1.66***	0.61	$-$ 2.74
$\beta _1 \, + {\, \beta _2^{5\%}}$	1.67	65.12	0.03	$-$ 6.35	262.80	$-$ 0.02
${\beta _1 \, +} {\, \beta _2^{5\%}}$ ${+ \, \beta _2^{2.5\%}}$	$-$ 14.88	66.83	$-$ 0.22	$-$ 8.41	253.05	$-$ 0.03
${\beta _1 \, +} {\, \beta _2^{5\%}}$ ${+ \, \beta _2^{2.5\%}}$ ${+ \, \beta _2^{1\%}}$	0.36	9.70	0.04	1.13	97.49	0.01
Swiss Franc						
$\beta _1$	$-$ 1.28***	0.28	$-$ 4.60	$-$ 1.67***	0.52	$-$ 3.23
$\beta _1 \, + {\, \beta _2^{5\%}}$	$-$ 11.31	11.93	$-$ 0.95	$-$ 54.24***	15.33	$-$ 3.54
${\beta _1 \, +} {\, \beta _2^{5\%}}$ ${+ \, \beta _2^{2.5\%}}$	$-$ 19.82	30.66	$-$ 0.65	42.86	110.50	0.39
${\beta _1 \, +} {\, \beta _2^{5\%}}$ ${+ \, \beta _2^{2.5\%}}$ ${+ \, \beta _2^{1\%}}$	2.42	5.97	0.41	3.04	9.41	0.32
Canadian Dollar						
$\beta _1$	$-$ 1.48***	0.53	$-$ 2.80	$-$ 1.27	0.81	$-$ 1.57
$\beta _1 \, + {\, \beta _2^{5\%}}$	$-$ 3.71	96.53	$-$ 0.04	91.14	266.69	0.34
${\beta _1 \, +} {\, \beta _2^{5\%}}$ ${+ \, \beta _2^{2.5\%}}$	58.60	75.41	0.78	24.65	298.01	0.08
${\beta _1 \, +} {\, \beta _2^{5\%}}$ ${+ \, \beta _2^{2.5\%}}$ ${+ \, \beta _2^{1\%}}$	$-$ 3.13	55.71	$-$ 0.06	12.69	142.04	0.09
Japanese Yen						
$\beta _1$	$-$ 0.79***	0.47	$-$ 1.70	$-$ 1.57**	0.74	$-$ 2.13
$\beta _1 \, + {\, \beta _2^{5\%}}$	$-$ 0.64	25.94	$-$ 0.02	$-$ 15.43	67.19	$-$ 0.23
${\beta _1 \, +} {\, \beta _2^{5\%}}$ ${+ \, \beta _2^{2.5\%}}$	27.74	31.86	0.87	55.32	66.88	0.83
${\beta _1 \, +} {\, \beta _2^{5\%}}$ ${+ \, \beta _2^{2.5\%}}$ ${+ \, \beta _2^{1\%}}$	9.77	9.57	1.02	2.29	51.12	0.04
Australian dollar						
$\beta _1$	$-$ 0.93***	0.33	$-$ 2.82	$-$ 1.31**	0.56	$-$ 2.35
$\beta _1 \, + {\, \beta _2^{5\%}}$	$-$ 6.72	28.22	$-$ 0.24	28.57	92.85	0.31
${\beta _1 \, +} {\, \beta _2^{5\%}}$ ${+ \, \beta _2^{2.5\%}}$	$-$ 61.96***	33.23	$-$ 1.86	187.10	117.52	1.59
${\beta _1 \, +} {\, \beta _2^{5\%}}$ ${+ \, \beta _2^{2.5\%}}$ ${+ \, \beta _2^{1\%}}$	$-$ 24.68	19.82	$-$ 1.24	13.92	68.11	0.20

This table reports coefficients associated with a regression model with GARCH error terms to assess the hedge and safe haven properties of Bitcoin

The estimated model is given by: $r_{btc,t} = \alpha + \beta _1 r_{c,t} + \beta _2^{5\%} r_{c,t} Q_5 + \beta _2^{2.5\%} r_{c,t} Q_{2.5} + \beta _2^{1\%} r_{c,t} Q_1 + \gamma ^{5\%} Q_5 + \gamma ^{2.5\%} Q_{2.5} + \gamma ^{1\%} Q_1 + e_t$ where $h_t = \omega + a e^2_{t-1} + b h_{t-1}$

The model is estimated for weekly and two-week data for the USD exchange rate to currencies, including the Euro (EUR), Pound Sterling (GBP), Swiss Franc (CHF), Canadian Dollar (CAD), Japanese Yen (JPY) and Australian Dollar (AUD)

***, ** and * indicate statistical significance at the 1%, 5% and 10% levels respectively

As with daily data, BTC is found to act as a strong hedge using weekly data for all currencies examined. At a two-week horizon, the strong hedging characteristics are only retained for the USD against GBP, CHF, YEN and AUD. This provides some initial guidance that any hedging properties will likely be horizon-dependent.

Assessing the safe haven properties of BTC for currencies, the coefficients on the downside risk interaction dummies are insignificantly different from zero in almost all cases. This again highlights that BTC is a weak safe haven for currencies. Some exceptions are notable. A significant negative coefficient is documented for the AUD at the 2.5th percentile at a one-week horizon. Likewise, a significant negative coefficient is observed at the 5th percentile for CHF. While these point to strong, safe haven properties, this should be interpreted carefully. For both exchange rates, these safe haven properties are not found at the other horizons examined, and they are only evident at a specific percentile, with findings not carrying over to the adjacent quantiles investigated.

Our findings concerning BTC’s long-horizon benefits are constrained by methodological limitations imposed by sub-sampling data to create longer horizons. Furthermore, this regression approach provides no insight into the points in calendar time that BTC acts as a hedge or safe haven. To further develop these analyses, we adopt an approach appropriate to capture the short-, medium- and long-run hedging capabilities at specific points in time.

### Wavelet coherency analysis

We next assess the relationships between currency returns and BTC simultaneously at various horizons (periods) and points in calendar time, using wavelet coherency. We first examine the entire period, 2010–2023, followed by a focus on the period surrounding and after the COVID-19 pandemic. The latter time period contains the market dislocation associated with the initial transmission of the virus internationally, the steep declines and subsequent rapid recovery in world equity markets, and the ensuant period of pervasive high inflation.

**Fig. 1:**
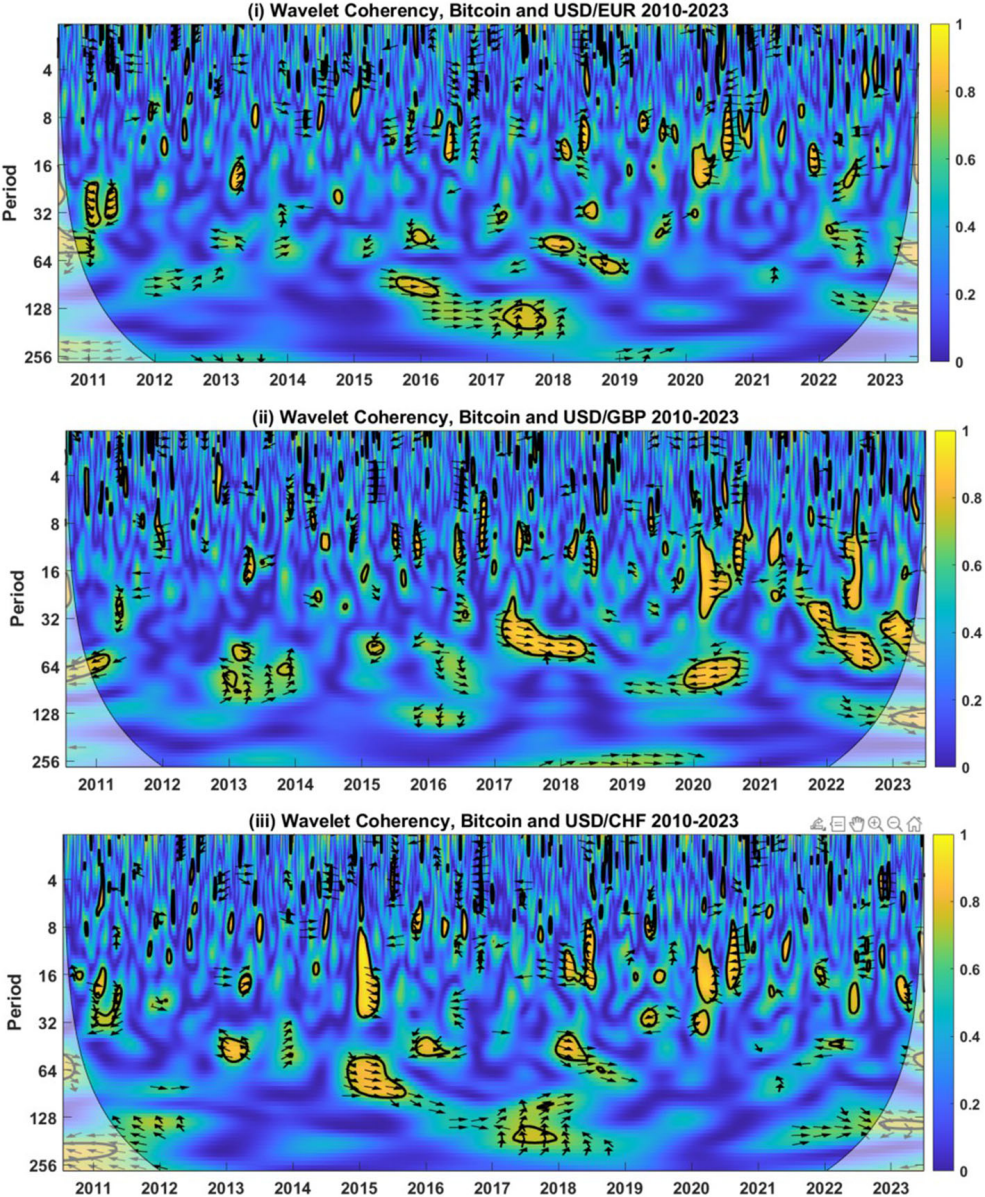
Wavelet Coherency Analysis of BTC and USD-GBP, USD-EUR, and USD-CHF exchange rates (2010–2023). Wavelet coherency between BTC and (i) the USD-EUR rate (ii) USD-GBP rate (iii) USD-CHF rate are shown. Calendar time is shown on the horizontal axis, while the period (horizon) is detailed on the vertical axis. A 5% significance level is estimated from the Monte Carlo simulation and represented by a thick black line. The regions influenced by border effects are denoted by the cone of influence, shown using a lighter black line. The regions of differing coherency are represented using a heat map ranging from blue (low coherency) to red (high coherency). East (west) facing arrows represent the in- (out-of-) phase, while north (south) facing arrows indicate that the currency leads (lags) BTC. A north-east (south-east) facing arrow symbolizes that the series are in-phase but that the currency (BTC) leads BTC (the currency). A northwest (south-west) facing arrow signifies that the series are out-of-phase but that BTC (the currency) leads the currency (BTC)

Figure 1 shows wavelet coherency between BTC and USD rates for the EUR, GBP, and AUD between 2010 and 2023. Regions with blue colouring indicate low coherency, representing a moderate or negligible interrelationship between BTC and the currency. Yellow bands surrounded by a thick black line indicate regions of high coherency that are significantly different from zero. Arrows denote the direction of the relationship in these bands and indicate any lead-lag relationships.

**Fig. 2:**
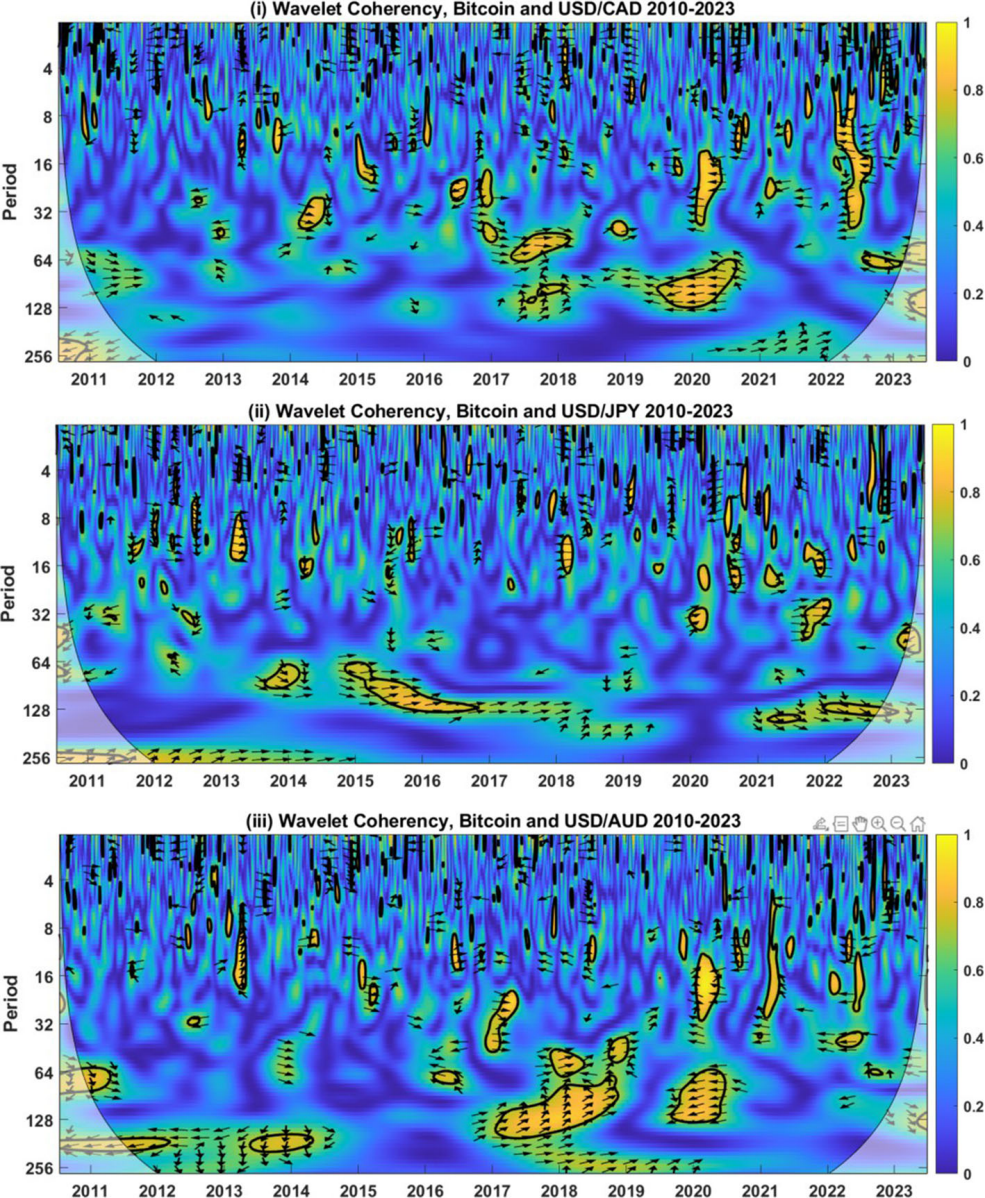
Wavelet Coherency Analysis of BTC and USD-CAD, USD-JPY, and USD-AUD exchange rates (2010–2023). Wavelet coherency between BTC and (i) the USD-CAD rate (ii) USD-JPY rate (iii) USD-AUD rate are shown. Calendar time is shown on the horizontal axis, while the period (horizon) is detailed on the vertical axis. A 5% significance level is estimated from Monte Carlo simulation and represented by a thick black line. The regions influenced by border effects are denoted by the cone of influence, shown using a lighter black line. The regions of differing coherency are represented using a heat map, which ranges from blue (low coherency) to red (high coherency). East (west) facing arrows represent the in- (out-of-) phase, while north (south) facing arrows indicate that the currency leads (lags) BTC. A north-east (south-east) facing arrow symbolizes that the series are in-phase but that the currency (BTC) leads BTC (the currency). A northwest (south-west) facing arrow signifies that the series are out-of-phase but that BTC (the currency) leads the currency (BTC)

For USD exchange rates with each of EUR, GBP, and AUD, we find only limited evidence of any strong relationships with BTC, which are primarily associated with long horizons. These limited relationships indicate that, on average, BTC acts as a weak hedge for the currencies considered. There is evidence, however, for some notable regions of high coherency. For example, for EUR, there are numerous medium- and long-run (between 8 and 180 days) regions, some of which have right-facing arrows, which indicates a positive relationship between the time series. For example, the region of positive coherency beginning in September 2015 at horizons between 80 and 100 days corresponds to an increase in the price of BTC alongside a concurrent strengthening of the USD relative to EUR. While this indicates a limit in the hedging possibilities at long horizons, diversification benefits from BTC may still be available to currency investors. There is also some evidence of negative relationships between BTC and the USD-EUR exchange rate, especially in the period surrounding the COVID-19 pandemic, something we assess in more detail shortly.

For the USD-GBP currency pair, while coherency is generally low (blue colouring), representing hedging opportunities, we observe some regions having notable levels of coherency. For example, from early 2017 to mid-2018, at periods of between 32 and 64 days, there is a patch of positive coherency. After the Brexit referendum in June 2016, GBP underwent a period of considerable volatility, losing value versus many currencies, including the USD. From January 2017, there was somewhat of a reversal in fortunes, with GBP increasing in value versus the USD. While BTC surged in price during 2017, there were periods during which both time series moved tightly in lockstep, especially during August and September, resulting in this long-horizon phase of positive coherency.

On January 15th 2015, the USD experienced a decrease in value relative to the CHF of $-17\%$ while BTC had dropped by $-42.5\%$ on the two days before this. This move in the USD-CHF rate resulted from the Swiss National Bank unexpectedly severing the peg of 1.20 CHF per EUR. During 2015, we observed two regions of positive coherency, one droplet for up to 32 days and a longer run region, both with southeast-facing phase arrows. This indicates a positive relationship with BTC leading CHF. In other words, an investor holding BTC as a hedge against the large draw-down for the USD-CHF rate would have experienced greater losses than if they had held the currency in isolation. BTC is not found to be a safe haven but instead increases investor risk during this time of currency market turmoil.

Figure 2 provides BTC wavelet coherency results for USD exchange rates with CAD, JPY, and CHF. As with the previous findings, the predominance of blue indicates low coherency on average, with the associated inference that BTC acts as a weak hedge for the currencies examined. Bands of high coherency are evident for each of the currencies, but the direction of the relationship varies over time. For example, the coherency between BTC and the USD-CAD rate indicates a positive and significant relationship between late 2016 and early 2018 at horizons ranging from 16 through 64 days. This indicates that BTC did not act as a hedge or safe haven for the USD-CAD rate during this time. West-facing arrows from 2020 indicate that returns from BTC and USD to CAD exchange rates were opposing during the COVID-19 pandemic, indicating potential strong, safe haven properties. We examine this more closely in the following Section.

For JPY, there are two significant long-run periods of high coherency beginning in mid-2013 and ending in 2017, where phase arrows are predominantly east-facing. This suggests that the series moves in tandem, pointing to a bound on the long-run safe haven properties. This incorporates the depreciation of the USD relative to JPY in June 2016, following the Brexit vote in the UK. Over the month, the USD depreciated 6.9% versus the JPY, including a day where it lost 3.76% (24th June 2016, the day following the Brexit vote). While there are doubts over the long-horizon safe haven properties, at short horizons, we find limited evidence of high coherency, indicating that BTC acts as a short-run hedge.

Finally, for the AUD, we detect several long-run regions of high coherency but with little consistency in the direction of the phase arrows. For example, in 2016, there was a band of higher coherency centred around periods of 64 days, with west-facing phase arrows. Over the first 6 months of 2016, the USD depreciated by 3.77% relative to the AUD, while the price of BTC increased by 45%, pointing to safe haven properties. During 2017–2018, both BTC and AUD were volatile. From 2017, there are two clear bands of coherency with east and northeast-facing phase arrows. A noteworthy concurrent decrease in BTC and the AUD is found between December 2017 and January 2018, when BTC lost over 51%, and the USD decreased by 5% versus AUD. Contrary to the experience in early 2016, this points to limited safe haven properties for BTC versus the USD-AUD rate.

These wavelet coherency-based findings indicate that BTC may act as a weak hedge for international currencies at short horizons but shed doubt over any long-horizon hedging and safe haven capabilities. Two findings stand out: BTC is occasionally a long-horizon hedge for currencies, but this relationship is inconsistent for all currencies over time and period. Second, a positive long-run relationship is often found between BTC and currencies, but this regularly corresponds to gains in the price of BTC occurring alongside an increase in the USD versus the currencies considered, indicative of diversification rather than hedging characteristics. As shown earlier, the statistical characteristics of BTC, such as volatility, skewness and tail risk, differ considerably from the currencies under examination. While BTC might theoretically meet the requirements to act as a currency hedge and safe haven, this mismatch in volatility, along with other distributional features, means that BTC cannot be a reliable safe haven for the USD.

**Fig. 3:**
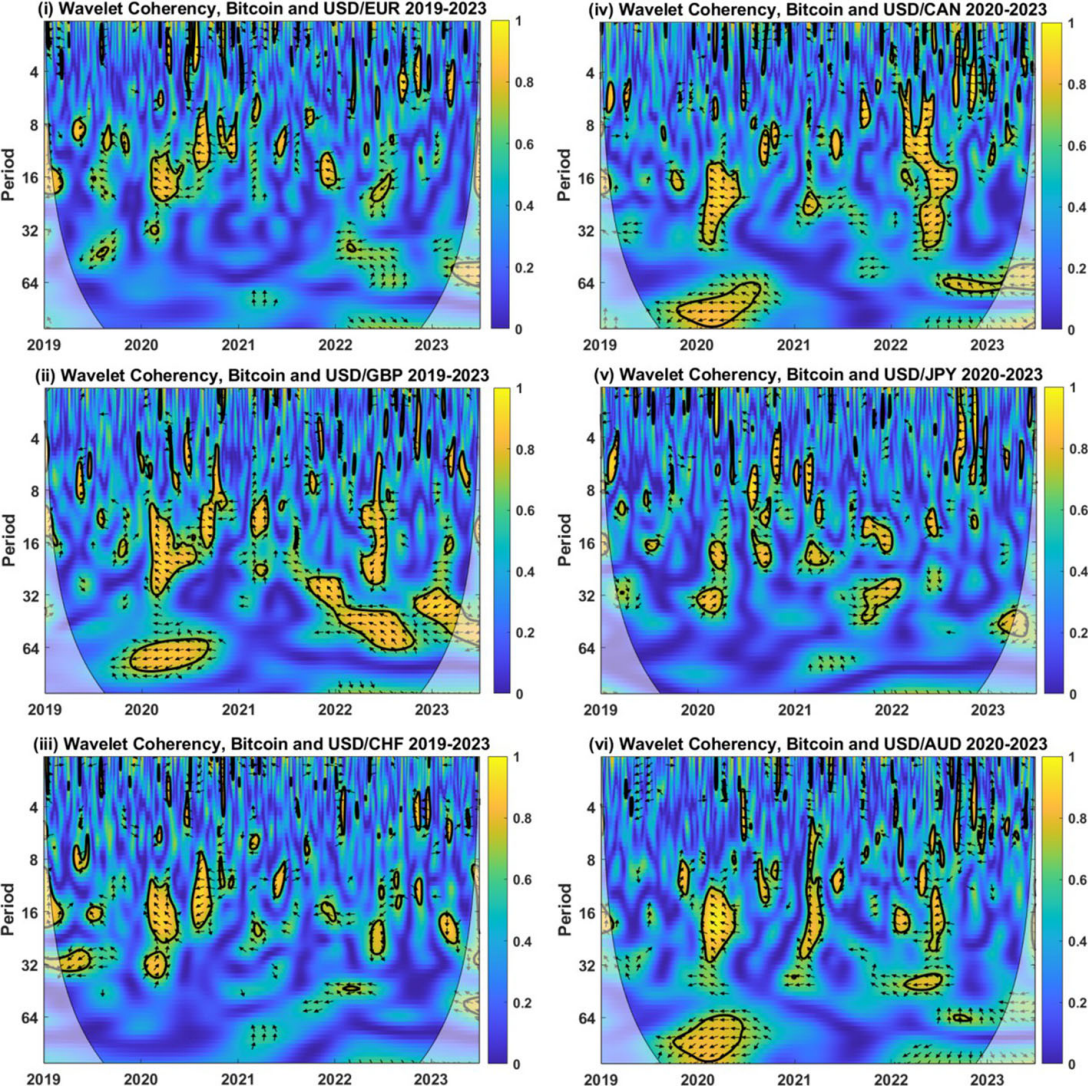
Wavelet Coherency Analysis of BTC and USD-EUR, USD-GBP, USD-CHF, USD-CAD, USD-JPY, and USD-AUD exchange rates over the period 2019–2023. Wavelet coherency between BTC and (i) the USD-EUR rate (ii) USD-GBP rate (iii) USD-CHF rate are shown between July 2019 and June 2020, encompassing the COVID-19 market turmoil. Calendar time is shown on the horizontal axis, while the period (horizon) is detailed on the vertical axis. A 5% significance level is estimated from Monte Carlo simulation and represented by a thick black line. The regions influenced by border effects are denoted by the cone of influence, shown using a lighter black line. The regions of differing coherency are represented using a heat map ranging from blue (low coherency) to red (high coherency). East (west) facing arrows represent the in- (out-of-) phase, while north (south) facing arrows indicate that the currency leads (lags) BTC. A north-east (south-east) facing arrow symbolizes that the series are in-phase but that the currency (BTC) leads BTC (the currency). A northwest (south-west) facing arrow signifies that the series are out-of-phase but that BTC (the currency) leads the currency (BTC)

As safe haven properties are of the greatest importance during periods of significant downside returns for the USD, we later investigate these relationships further using quantile coherency.

#### Wavelet coherency analysis surrounding the COVID-19 Pandemic

As highlighted in Table 1, the level of downside risk associated with currencies increased markedly during 2020, coinciding with the COVID-19 pandemic. In this section, we examine the level of wavelet coherency between BTC and currencies over the period January 2019 - June 2023, incorporating the initial market reaction to the pandemic, previously examined in the context of equity markets (Conlon & McGee, 2020; Conlon et al., 2020), along with the ensuant period of inflationary pressures (Blau et al., 2021; Conlon et al., 2021).

Results are shown in Fig. 3. Although phases of low coherency (blue colour) remain dominant, the evidence is weaker than found over the entire 2010–2023 period. This suggests that the hedging properties of BTC may not be consistently upheld throughout this period, but to confirm this, we must look at the phase arrows relating to the zones of high coherency.

For each of the currencies, we find an extended band of high coherency beginning in February 2020 and spanning horizons of 8 to 32 days. The northwest-facing phase arrows indicate that BTC has a negative relationship with each of the currencies and that changes in BTC lead to currency movements. However, this negative interrelationship is not an indicator of hedging or safe haven properties. Over the first 15 trading days of March, BTC fell in price by $34.87\%$ while the USD appreciated substantially. For example, the USD strengthened $3.05\%$, $9.60\%$ and $2.17\%$ against the EUR, GBP, and CHF, respectively. Currency hedging using BTC during this period would potentially have resulted in large losses for investors, depending upon the hedge ratio employed.

Findings for JPY are more intricate. For longer periods of 32 days or more, we find a positive coherency stretching from January to April 2020. At shorter horizons, this effect is reversed, with evidence for negative coherency at horizons between 16 and 24 days. A further droplet, displaying positive coherency, is focused on March 2020 for periods of up to 4 days. The latter corresponds to the height of the market reaction to the COVID-19 pandemic. The JPY, itself a safe haven, strengthens against the USD, resulting in concurrent losses and this evident positive relationship. These findings suggest that BTC is also not a hedge or safe haven for the USD-JPY currency pair during the COVID-19 crisis, a function of the previously documented evidence that JPY acts as a safe haven in itself for USD (Ranaldo & Söderlind, 2010).

Shortly after the onset of COVID-19, the US consumer price index, representing the rate of inflation, began to rise. From this point, we find some evidence for bands of negative coherency across all currencies. These are most noteworthy for the USD-CAD and USD-GBP currency pairs during 2022. Negative coherency, in this case, does not, however, correspond to safe haven properties. The price of Bitcoin fell by 64% in 2022, accompanied by a strengthening of the CAD and GBP with respect to the USD. In other words, a USD investor intending to use BTC as a hedge during this period would have borne significant losses from the hedging asset. Similar findings are evident for the other currencies examined.

The wavelet transformation allows us to discern the timing and strength of relationships between BTC and currencies. While the earlier finding that BTC acts as a hedge on average for the USD is reiterated, this may come at a cost. During the COVID pandemic, holding a position in BTC would have resulted in large losses for investors. Moreover, in the high inflationary period which emerged after the pandemic, USD investors looking to BTC to hedge downside risk would have encountered large negative price movements. While these do not correspond to periods of large USD losses, they do highlight that the volatility inherent in BTC makes it a poor hedging instrument for currency investors.

### Quantile coherency

Wavelet coherency allows us to determine the hedging capacity of BTC for currencies and indicates the relationships at particular horizons and points in time. Additional analysis is, however, required to conclusively establish whether BTC acts as a safe haven at different frequencies. The quantile coherency approach proposed by Baruník and Kley (2019) and detailed in Sect. 2.3 is used to simultaneously understand the short- and long-run relationships between BTC and currencies during periods where the USD experiences its most extreme negative returns. In contrast to the wavelet approach detailed previously, this method focuses on specific quantiles, allowing us to better quantify the safe haven properties of BTC for each currency. If BTC is a strong safe haven, we expect to find a negative interrelationship during such extreme currency moves. If the relationship is insignificant, this would indicate that Bitcoin acts as a weak safe haven.

**Fig. 4:**
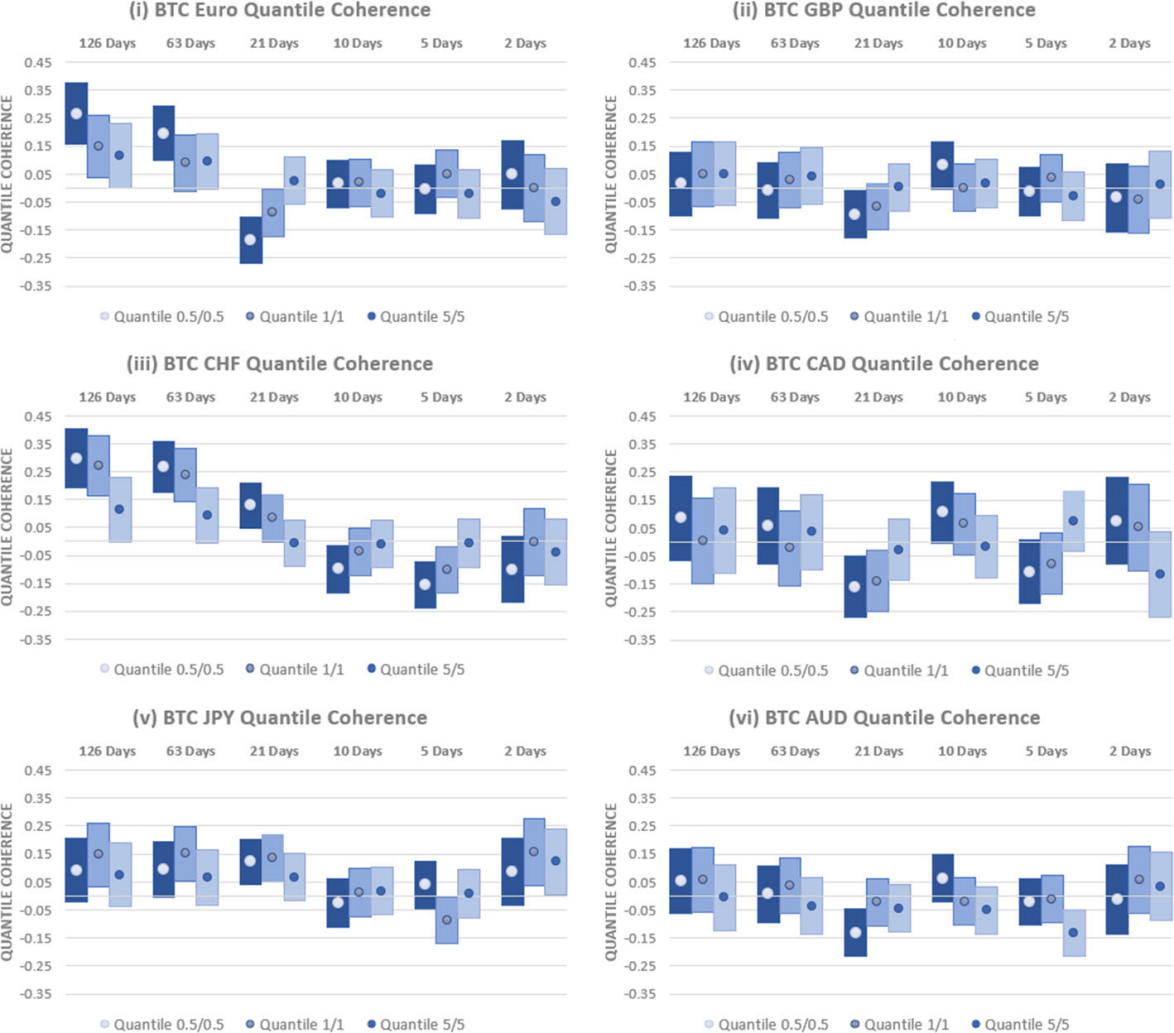
Quantile Coherency Analysis of BTC and Currencies (2010–2023). Quantile coherency estimates between BTC and the (i) USD-EUR (ii) USD-GBP (iii) USD-CHF (iv) USD-CAD (v) USD-JPY and (vi) USD-AUD rates between 2010 and 2020 are shown. Quantile coherency for 0.5%, 1% and 5% quantiles are shown at horizons ranging from 2 days through 126 days. 95% confidence intervals are shown using bar plots, and significance is indicated where bar plots do not overlap with the x-axis

Results are shown in Fig. 4 for the $0.5\%$, $1\%$ and $5\%$ quantiles across the currencies examined over the full period examined, 2010–2023. Considering first the relationship between USD-EUR and BTC, we find significant differences between short- and long-run results. At the shortest horizons, the level of quantile coherency is negligible, with $95\%$ confidence intervals that indicate coherency estimates that are not significantly different from zero. At intermediate horizons, only limited evidence of a significant relationship is evident. At a 21-day horizon, a negative and significant coherency is found at the 0.5th percentile. This indicates that Bitcoin acts as a safe haven, but this should be interpreted carefully given the lack of supporting evidence at other horizons or quantiles. At the longest horizons considered, 63 days and 126 days, approximately 3 and 6 months, a positive relationship between BTC and the USD-EUR exchange rate is found. This relationship is statistically different from zero for the 0.5th percentile at both horizons and for the 1st percentile at the 63-day horizon. These findings demonstrate that BTC is a weak short-horizon safe haven for the USD-EUR rate, but, at long horizons, rather than acting as a safe haven, it would result in increased losses when held alongside EUR. Our results contrast with those of Baumöhl (2019), where a negative quantile relationship was documented between BTC and USD-EUR at long horizons. This distinction may be attributable to the longer data series incorporating the COVID crisis employed in this study.

Similarly, we find no evidence that BTC acts as a strong safe haven for any of the other currencies considered. For USD-GBP, BTC is a weak safe haven, as indicated by a coherency that is close to zero. Similar to findings for USD-EUR, the only exception is at a 21-day horizon, where a negative and significant relationship with BTC is found at the lowest quantile examined. As with the EUR, this does not persist across other horizons or quantiles and, so, provides limited value to investors.

Results for the USD-CHF rate are very similar to those obtained for EUR. At short- to intermediate horizons, there is some evidence of a negative relationship with BTC at the lowest quantiles examined. However, this may come at a cost as a positive and significant relationship is found at the longest horizons examined, pointing to concurrent losses between BTC and the USD-CHF rate. Again, rather than acting as a safe haven for CHF, holding a position in BTC results in increased losses relative to holding the currency in isolation. The relationships between BTC and the USD exchange rate with CAD and AUD are generally negligible. Only at a 21-day horizon do we find evidence of a significant (negative) relationship, indicating that BTC acts as a weak safe haven for both currencies during periods of significant downside risk.

Finally, we assess the capacity of BTC to act as a safe haven for the USD-JPY rate. Across all horizons and quantiles, there is no evidence of any negative relationships with BTC, ruling out any strong safe haven characteristics. Moreover, there is both short- and long-horizon evidence for a positive relationship, indicating that BTC does not provide any consistent, safe haven benefits for USD investors worrying about depreciation relative to the JPY.

**Fig. 5:**
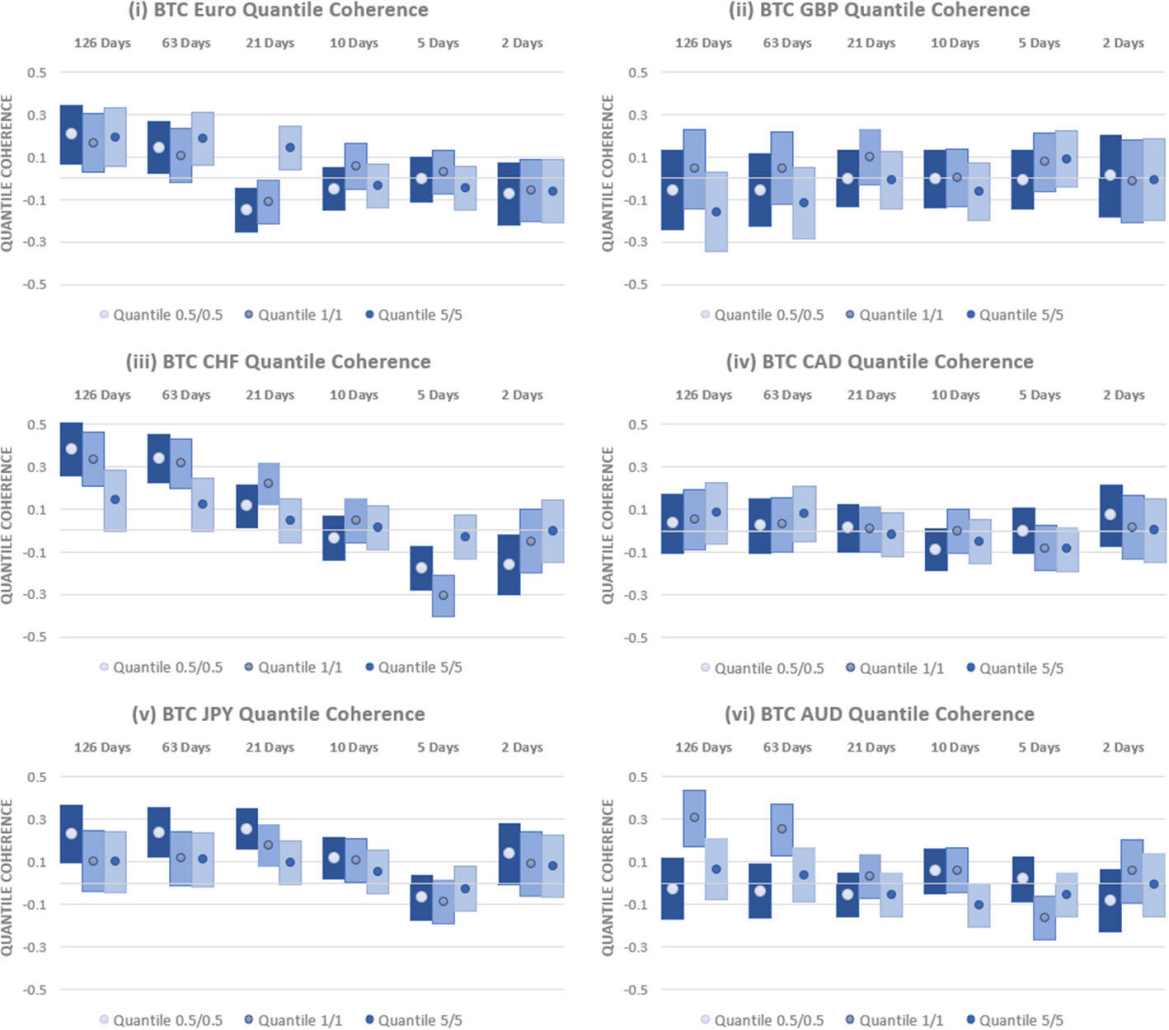
Quantile Coherency Analysis of BTC and Currencies (2016–2023). Quantile coherency estimates between BTC and the (i) USD-EUR (ii) USD-GBP (iii) USD-CHF (iv) USD-CAD (v) USD-JPY and (vi) USD-AUD rates between 2010 and 2020 are shown. Quantile coherency for 0.5%, 1% and 5% quantiles are shown at horizons ranging from 2 days through 126 days. 95% confidence intervals are shown using bar plots and significance is indicated where bar plots do not overlap with the x-axis

We next assess how these results are impacted by the relatively low trading volume and high volatility in BTC in the years after its launch. To this end, we examine the quantile coherency between BTC and each currency over the period 2016–2023. Results are provided in Fig. 5. Inference is generally unaltered. BTC has a positive relationship with USD-EUR at long horizons, pointing to the potential for losses if used as a hedging instrument. Although some evidence of a negative relationship exists at a 21-day horizon, the central takeaway is that BTC is, at best, a short-horizon weak safe haven for USD-EUR investors. No significant coherency relationships are found for USD-GBP and USD-CAD, which also points to weak safe haven properties.

USD-CHF displays a positive relationship with BTC at the longest horizons examined, reducing to a negative relationship at a 5-day horizon. This emphasises the short-horizon safe haven properties but with a substantial risk of large losses in both the USD-CHF rate and BTC for longer-term investors. Similar uncertainty surrounds the safe haven characteristics of BTC for USD-JPY and USD-CAD. While there is some support for short-horizon safe haven properties, these are balanced by evidence for a positive relationship and concurrent losses at longer horizons.

**Fig. 6:**
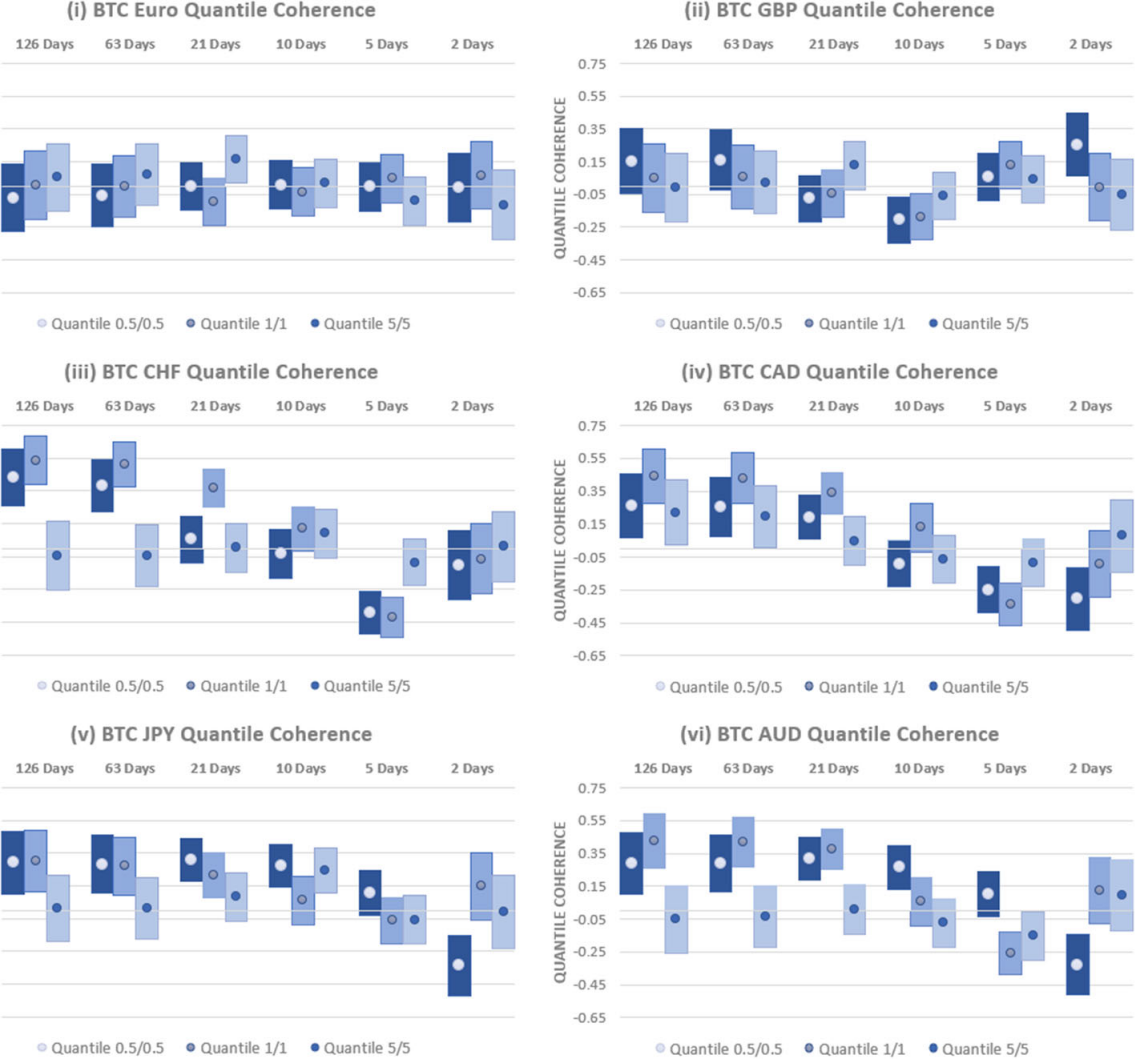
Quantile Coherency Analysis of BTC and Currencies (2020–2023). Quantile coherency estimates between BTC and the (i) USD-EUR (ii) USD-GBP (iii) USD-CHF (iv) USD-CAD (v) USD-JPY and (vi) USD-AUD rates between 2010 and 2020 are shown. Quantile coherency for 0.5%, 1% and 5% quantiles are shown at horizons ranging from 2 days through 126 days. 95% confidence intervals are shown using bar plots and significance is indicated where bar plots do not overlap with the x-axis

Next, in order to isolate the safe haven properties during a period of substantial inflationary pressures, we examine the quantile coherency relationships between July 2020 and June 2023. Results, presented in Fig. 6, again highlight that BTC is, at best a weak safe haven for currencies, with potential for long-run losses. The relationship between USD-EUR and BTC is insignificant across all horizons and quantiles, indicating weak safe haven properties. Findings for GBP are similar at long horizons but with some evidence of both strong, safe haven properties and concurrent relationships at shorter horizons, placing doubt over the reliability of any hedging capabilities.

For the USD rate versus CHF, CAD, JPY and AUD, there is strong evidence of a positive relationship with BTC at low quantiles from horizons as short as 10 days and longer. While there is some evidence for negative links and, thus, strong, safe haven properties at short horizons, hedging using BTC once more comes at a long-horizon cost.

**Fig. 7:**
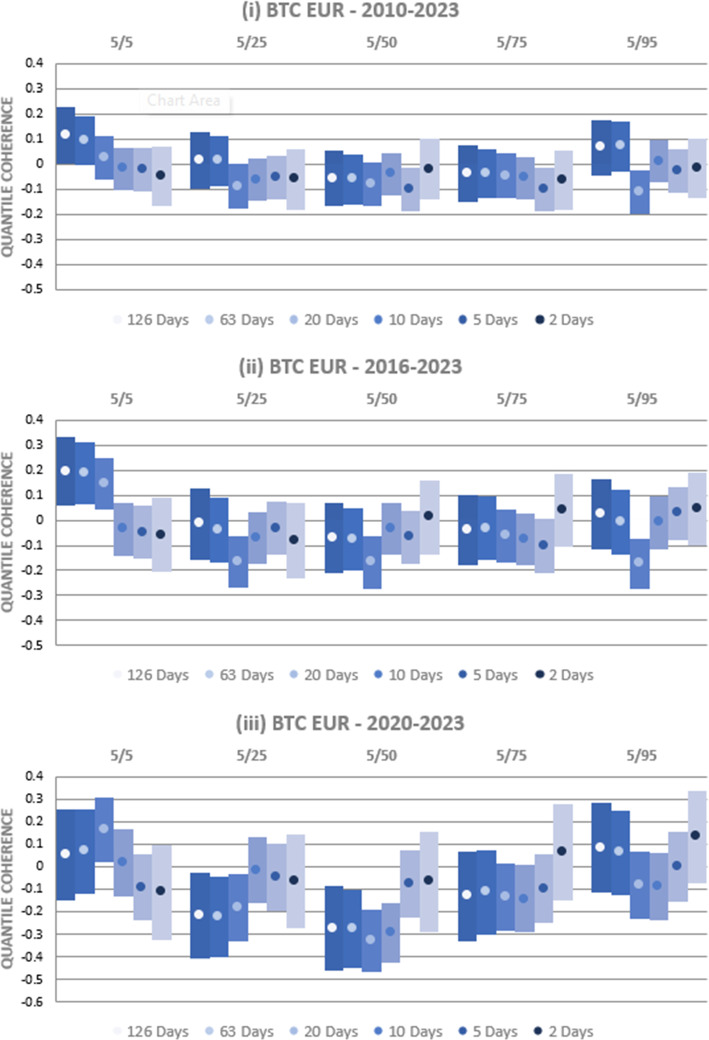
Quantile Coherency Analysis of BTC and USD-EUR at different quantiles (2010–2023). Quantile coherency estimates between BTC and the USD-EUR rates between 2010 and 2020 are shown. While the USD is constrained to the 5% quantile, BTC is examined at the 5%, 25%, 50%, 75% and 95% quantiles at horizons ranging from 2 days through 126 days. 95% confidence intervals are shown using bar plots, and significance is indicated where bar plots do not overlap with the x-axis

In Fig. 7, we examine a range of alternative BTC quantiles across the three time periods previously assessed while retaining the 5th percentile as a baseline for the USD-EUR rate. In other words, when losses for the USD exchange rate with EUR are significant, does the interrelationship with BTC depend upon whether BTC also has extreme current losses? The findings are clear over the 2010–2023 period; for higher BTC quantiles, we find no consistent evidence of any significant relationships between BTC and EUR, pointing to weak safe haven properties. Over the 2016–2023 period, we replicated the earlier findings at the lowest quantile examined, 5/5, with a significant positive relationship at long horizons. At other quantiles, there is, at best, limited evidence of any significant coherency. Finally, negative coherency is evident over the most recent 2020–2023 period at long horizons when BTC is at intermediate quantiles. This implies that, during intense inflationary pressures, BTC acted as a strong safe haven, but only when its returns were not at their most extreme. Beyond these limited criteria, BTC is again found to be a weak safe haven, exhibiting no significant coherency. This analysis of additional quantiles highlights that BTC is predominantly a weak safe haven for the USD-EUR currency rate but with the potential for long-horizon positive coherency at the extremes. This resonates with our earlier findings that, while BTC may offer some hedging benefits for currency investors, these are compromised by the potential for large concurrent losses at extremes.

## Conclusion

The potential for BTC to act as a hedge or safe haven for equity markets has received considerable attention. Less well understood are the interrelationships between BTC and currency markets and any hedging and safe haven properties that might emerge. Unlike traditional fiat currencies, BTC cannot be devalued through inflation and is not linked to monetary policies, motivating its use as a currency risk management tool.

In this paper, we employ three different horizon-dependent methodologies to isolate any hedging or safe haven properties of BTC for the US dollar. A non-linear regression approach sheds some light on the research question, but data availability limits the analysis to short- and medium-run horizons. Wavelet analysis allows us to simultaneously determine the points in time and the horizons at which BTC acts as a hedge or safe haven. Quantile coherency analysis isolates BTC’s safe haven properties during extreme losses for the US dollar. The ability of these methodologies to shed light on the horizon-dependent links between BTC and USD builds upon previous literature, highlighting the importance of examining horizon-dependent financial characteristics.

Our findings indicate that BTC meets popular test criteria in the literature to be considered a strong hedge for the US dollar at short time horizons. However, BTC is found to be a weak short-horizon safe haven for most currencies, displaying a limited dependency during times of currency market turmoil. Finally, for long-run horizons, BTC is found to have a significant positive dependency with large negative price movements in a range of currencies. This indicates that rather than providing safe haven benefits, hedging using BTC would result in increased risk.

While this is not the first paper to consider the hedging and safe haven properties of BTC for currencies, we build upon the nascent literature and provide new insights relevant to investors. Our analysis of the BTC’s hedging and safe haven characteristics during the COVID-19 crisis expands our appreciation of this new asset to a significant crisis period. The methodologies employed also allow us to identify long-run properties not considered in previous literature and relevant to the marginal investor. The results detailed in this research indicate that, while BTC may have some weak hedging and safe haven attributes for the US dollar, this may be at the price of long-run concurrent losses during periods of acute depreciation in the US dollar. From the perspective of an investor, the extreme volatility of bitcoin, alongside other distributional mismatches, makes BTC a poor risk management tool for currencies. While there are occasional periods where BTC possesses hedging properties, a hedging instrument that has the potential to result in large losses at exactly the times investors are seeking respite from currency volatility fails to meet the criteria for a safe haven.

A limitation of this research is the short available price history for BTC. For gold, bonds and other plausible safe haven assets, a lengthy history, at least back to 1968 (Conlon et al., 2018b), is available, allowing for a thorough analysis of hedging and safe haven behaviour throughout multiple market cycles. In contrast, BTC has only existed since 2010 and encountered only one period of serious dislocation across all markets in 2020. Future research might also assess the hedging and safe haven capacity of other cryptocurrencies for the USD. While BTC is representative of the cryptocurrency market, there is an abundance of other digital currencies such as stablecoins, altcoins, memecoins and non-fungible tokens (NFTs). Among the almost 8,800 currently traded coins (as of January 2024), many others may have stronger and more reliable hedging and safe haven properties for the USD than BTC.

## Footnotes

1 It has been suggested, however, that, with sufficient agreement among users, the algorithm controlling the supply of BTC could be adjusted, perhaps presenting a tail risk limiting its potential as a currency hedge.

2 For a comprehensive review of the role of gold as an investment asset, see O’Connor et al. (2015).

3 Goodell (2020) provides an outline of the economic consequences associated with the COVID-19 and implications for financial markets.

4 We expand on several recent works that focus on the effects of the pandemic on contagion (Corbet et al., 2020) and (Corbet et al., 2020b).

5 Some examples of the use of wavelet analysis in a financial context include the assessment of diversification benefits from investing in international markets (Berger & Gençay, 2020; Conlon et al., 2018a; Rua & Nunes, 2009) links between commodity markets and other asset classes, and estimation of stock systematic risk at different scales (Gençay et al., 2005). Gençay et al. (2001) and Percival and Walden (2000) provide a detailed technical treatise of wavelet multi-scale analysis.

6 See, for example, Goodell and Goutte (2020), Bekiros et al. (2017); Bredin et al. (2015) and Rua and Nunes (2009).

7 Other wavelet basis functions were also tested without any qualitative changes to findings.

8 The literature on wavelets equivalently refers to the scale, period and horizon associated with the decomposition, each of which may be interpreted as a form of the wavelength. The inverse of the wavelength is the frequency, measuring the number of cycles per period.

9 This analysis can be distinguished from previous work where quantile approaches were employed but without an explicit focus on the frequency or horizon properties associated with BTC interdependencies. See, for example, Chevapatrakul and Mascia (2019).

10 Source: Triennial Central Bank Survey OTC foreign exchange turnover in April 2022. We note that the Chinese Renminbi is not examined as, while this currency is not pegged to the US Dollar, it traded within a small range over the period examined.
